# Effects of ZnO/trimethylsilyl cellulose nano-composite coating on anti-UV and anti-fungal properties of papers

**DOI:** 10.1038/s41598-023-45853-2

**Published:** 2023-11-24

**Authors:** Jaroenporn Chokboribal, Lunjakorn Amornkitbamrung, Wisawakorn Somchit, Voravadee Suchaiya, Pemika Khamweera, Piyapong Pankaew

**Affiliations:** 1 Materials Science Program, Phranakhon Rajabhat University, Bangkok, Thailand; 2 Sensor Research Unit, Department of Chemistry, Chulalongkorn University, Bangkok, Thailand; 3 Product Innovation and Technology Program, Phranakhon Rajabhat University, Bangkok, Thailand; 4 Division of Industrial Materials Science, Rajamangala University of Technology Phra Nakhon, Bangkok, Thailand

**Keywords:** Materials science, Nanoscience and technology

## Abstract

Trimethylsilyl cellulose (TMSC) was employed as the coating matrix for the application of zinc oxide nanoparticles (ZnO) onto paper surfaces and the protections of ZnO/TMSC coating against UV-induced damages and fungal spoilage were evaluated. Filter papers were immersed in 2% w/v TMSC solution loaded with ZnO and air-dried. Three ZnO/TMSC suspensions were prepared with 0.1, 0.5, and 1% w/v ZnO NPs. The presences of ZnO/TMSC protective layers were confirmed with ATR-IR spectroscopy. The coated papers exhibited high surface hydrophobicities. After the coated papers were subject to 365-nm UV irradiation at 400 W for 3 h, the contact angles dramatically dropped. The trimethylsilyl (TMS) groups exposed on the surface formed a moisture barrier and were partially removed on UV exposure. ATR-IR revealed that more TMS groups were removed in the protective layer with no ZnO. UV-irradiated papers turned yellow and papers protected with 1% ZnO/TMSC exhibited significantly lower color changes than that of the uncoated one. Compared to the TMSC-coated paper, the addition of ZnO resulted in a significant reduction in tensile strength at maximum. However, after UV irradiation, significant increases in both the strain at break and strength at maximum were only observed in 1% ZnO/TMSC-protected papers. Regarding their anti-fungal properties, the 1% ZnO/TMSC films were effective in growth inhibitions of *Aspergillu*s sp. and *Penicillium* sp. on the nonirradiated papers. Despite being hydrophilic after UV-irradiation, growths of the molds were severely suppressed on the UV-irradiated paper.

## Introduction

As an alternative that predates digital media, paper is a light, low-cost, and readily available material for recording history and data and the disseminations of information, knowledges, and written artistic works. It is the medium of artworks and picture/photograph reproductions. It is biodegradable and can be produced in a sustainable process. Combining the ability to carry information (texts and artworks) and its foldability and lightness, paper is also a common material used as a product package^1^.

Paper is classified as a bio-based material because the main component is cellulose, a polysaccharide derived from plants. However, being a natural-based material, paper is susceptible to degradation. Various factors can cause paper deterioration, such as mechanical forces (cutting and tearing) and chemical degradation (moisture, acid hydrolysis, and alkaline degradation). Additionally, exposure to light (ultraviolet radiation) and invasion by microorganisms (such as mold and bacteria) are other major factors that contribute to paper deterioration^2^.

The long-wave region (315–400 nm) of ultraviolet (UV) radiations is called UV-A region. The UV-A accounts for about approximately 95% UV radiation reaching the Earth surface^3^. Prolonged exposure to UV radiation, from natural or artificial light sources, causes a serious deterioration in paper structure that culminates in the yellowing and strength loss of paper^4^. The deterioration of papers is of concerns, especially in the case of paper-based food packages in which damages in the cellulose structures impaired the packages’ abilities to protect food from ambient moisture, oxidation with oxygen in air, or even microbial spoilages^5^. Such processes result in losses in sensorial quality of foods such as reduced crispiness, undesirable smells (bad or rancid odor), altered taste, changes in color, and losses of nutritional values. The foods are considered stale by the customers and their shelf lives are shortened as a result. The study of methods to mitigate the effects of UV exposure is crucial for the development of cellulose-based materials exhibiting high UV resistance.

Papers can undergo degradation under UV radiation through a process and mechanism that involves the oxidation of glucose residues of cellulose chains and radical formation^6^. The photo-oxidation of cellulose can be attributed to carbonyl and carboxyl groups, which are known initiators of photochemical reactions^7^. During UV exposure, oxidation is associated with the release of free radicals. Free radicals are highly reactive and can destroy and depolymerize cellulose and its derivatives through radical chain reactions. This can result in the yellowing and fragility of papers^8^.

The deterioration of paper by microorganisms, such as bacteria and fungi, particularly filamentous fungi (mold), can occur easily in indoor environments with sufficient moisture^9^. Mold grows and reproduces through digestive enzymes and spores, resulting in stains on paper-based items like documents and books^10^. The presence of mold contamination not only poses a serious risk to collections of documents and vital records, potentially causing significant damage but also to human health since mold spores can enter the body through inhalation. When mold is active, it can spread rapidly and is more likely to cause allergic or respiratory symptoms^11^.

To develop a material to protect paper from photo-oxidation and microorganisms at the same time, ZnO is a promising material, owing to its low cost, high availability, and useful chemical and optical properties. ZnO is mostly used in the form of nanomaterials (NPs), which can be synthesized through precipitation and hydrothermal approaches^12–14^. ZnO has excellent semiconducting properties with a direct band gap of 3.37 eV and an exciton binding energy of 60 MeV at room temperature. The semiconducting properties of ZnO make it an effective UV absorber. Previous studies have reported the utilization of ZnO nanomaterial doping on cotton fabrics for UV-blocking properties^15,16^. Additionally, the gap between conduction and valence electrons plays an important role in the formation of reactive oxygen species which in turn can destroy microbial cell membranes^17^. ZnO also exhibits antimicrobial activity due to the electron pairs generated through photo-oxidation and reduction processes^18,19^. In addition to protecting paper from degradation, it is important to address the issue of paper strength which can diminish over time. Thus, reinforcing the strength of paper is a crucial consideration. Particularly, cellulose-based materials have been used extensively for paper strengthening due to their suitable compatibility with paper. Coating papers with a cellulose derivative, such as carboxymethyl cellulose (CMC) or carboxyethyl cellulose (CEC) in ethanol, can increase the strength and service lives of paper^20,21^. An aqueous solution of methylcellulose (MC) can also be applied for paper strengthening^22–24^. Recently, solution of organo-soluble cellulose derivative has been proposed as a method for strengthening paper. This solution offers the advantage of not significantly altering the stability of the paper after treatment since cellulose fibers have a very low swelling capacity in this solvent. For instance, a solution of trimethylsilyl cellulose (TMSC) in hexamethyldisiloxane (HMDSO) can improve paper strength by 20%^25,26^.

**Figure 1:**
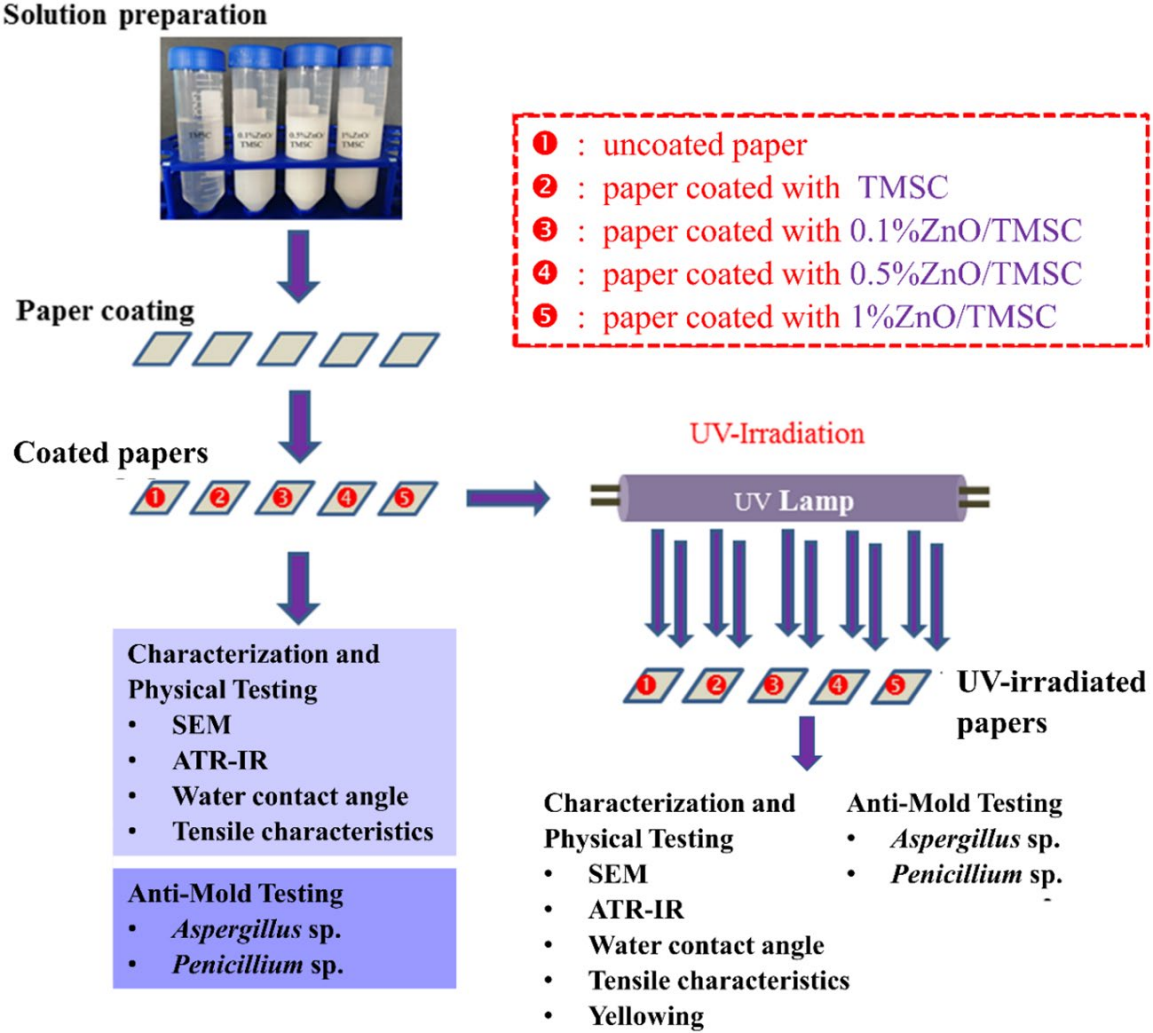
Schematic summary of the methodology and results of the present study.

In this contribution, the authors have developed a nano-composite coating of ZnO NPs and TMSC and studied the effects of the films on the anti-UV and anti-fungal properties of the coated papers. The ultimate goal is to exploit the anti-UV, anti-microbial, and moisture-repelling properties of the ZnO/TMSC coating in the manufacturing of robust and durable paper-based packages for foods and portable electronic devices. ZnO NPs acted as a UV-absorber and an anti-microbial agent, whereas TMSC acted as astrengthening agent for the paper and as a stabilizer to evenly distribute ZnO NPs in the coating films. Several techniques were employed to determine the protective efficacies of the coating against UV-induced damages. Tensile characteristics, water contact angles, and yellowing were investigated. ATR-IR spectra provided the clue on how the TMSC and ZnO/TMSC coating were affected by UV irradiation. Finally, the anti-fungal properties of the coating were evaluated by observing the growth of selected molds on the coated papers. The methodology and overview of the present study are summarized in Fig. 1.

## Materials and methods

### Materials

Filter papers (Johnson grade 304, pore size 5‒13 µm, diameter 125 mm) were purchased from Unitywewell Co., Ltd. (Thailand). Zinc oxide nanoparticles (ZnO NPs, 99%, 35‒45 nm) were purchased from US Research Nanomaterials, Inc. (USA). Trimethylsilyl cellulose (TMSC, degree of substitution 2.8, molecular weight 149,000 g /mol, derived from Avicel PH-101) and hexamethyldisiloxane (HMDSO, 98%) was obtained from TITK (Thüringisches Institut für Textil- und Kunststoff-Forschung e.V., Rudolstadt, Germany). and Acros Organics (Germany), respectively. Penicillin/streptomycin and potato dextrose agar (PDA) were obtained from Lab System Co., Ltd., Thailand. All chemicals were used as received without purification.

### Filter papers dip-coating

In HMDSO, 2% w/v TMSC solution was first prepared. To the continuously stirred 2% w/v TMSC solution maintained at 50 °C, ZnO NPs were gradually added and the suspension was kept stirred for 2 h. The ZnO/TMSC suspensions were then ultrasonicated at room temperature for a further 30 min using an Elmasonic S40 device (340 W, Elma, Germany). Three ZnO/TMSC suspensions were prepared with 0.1, 0.5, and 1% w/v ZnO NPs.

Filter papers were cut into a square of 2 × 2 cm^2^ and immersed in the ZnO/TMSC colloidal suspension for 5 min and allowed to dry at room temperature for 5 min.

### UV irradiation of papers

UV irradiations were carried out inside a 80 × 60 × 50-cm^3^ wooden box lined with aluminum foil. The papers were placed on a stage at a fixed distance of 15 cm from the UV lamp and exposed to 365-nm UV light at an intensity of 400 W for 3 h.

### Characterizations

The UV–visible absorptions (200‒700 nm, 200 nm/min) were analyzed using the Thermo Evolution 201 UV–Vis Spectrophotometer.

Attenuated total reflection infrared (ATR-IR) spectra (4000‒400 cm^‒1^, 32 scans at a resolution of 4 cm^‒1^) were recorded using the Nicolet iS5 Fourier transform infrared (FT-IR) spectrometer equipped with an iD7 ATR accessory (Thermo Fisher Scientific Inc., Waltham, MA, USA).

Scanning electron microscopic (SEM) images were taken using the JSM-6510A microscope (JEOL Ltd., Tokyo, Japan) operated at 10 kV. All samples were sputter-coated with gold.

Images of a 3-µL droplet of deionized water on the sample surface were taken after 5 s using the DMe-210 contact angle meter (Kyowa, Japan). The contact angles were determined from the images using the FAMAS software (*n* = 5).

CIE L*, a*, b* color coordinates of the papers were determined (*n* = 3) using the Color Muse colorimeter (Illuminant D50, aperture size 4 mm, angle of 2°, measurement geometry 45/0). This low-cost colorimeter was shown by Dang et al.^27^ to be a precise instrument for detecting relative color differences. The total color changes (ΔE*) were determined according to Eq. (1), where ΔL*, Δa* and Δb*are the differences of the respective values of the same sample measured before and after UV irradiation^28,29^.

1 $${\Delta E}^{*} = \sqrt{{{(\Delta L}^{*})}^{2}+{{(\Delta a}^{*})}^{2}+{{(\Delta b}^{*})}^{2}}$$

The tensile strength at maximum and strain at break of the papers were determined according to the standard method TAPPI T494 om-01 using the Instron 5966 universal testing machine. Strips of papers (4 × 50 mm^2^) were vertically mounted with two clamps located 2.5 cm apart. Sample stretching was carried out at 1 mm/min using a 5-kN load cell. At least ten experimental runs were carried out for each condition. All papers were conditioned at 23 °C and 65% relative humidity for 24 h before the test.

### Statistical analyses

All data were collected and presented as mean ± standard error. Group comparisons were performed using one-way ANOVA and Duncan’s multiple range test (DMRT) using the SPSS program version 25. Significance was assumed at α = 0.05. Conditions sharing the same DMRT post hoc letter were not significantly different.

### Determinations of anti-fungal activities

Fungal samples were collected from a book. Paper pages, spinal area, and inside of front and back covers of the book were wiped with sterile cotton swabs in three directions (horizontal, vertical, and diagonal). The swab heads were extracted with NaCl solution (0.1%) in sterile tubes. The solutions were spread on PDA medium and incubated at 30 ± 2 °C for 48 h. The colony characteristics and morphologies of reproductive structures were examined for fungal screening.

Inocula of the selected fungal species were evenly spread throughout the surface of PDA. Circular paper samples with a diameter of ~ 6 mm were placed over the spread inocula and pressed down lightly to ensure good contacts. The distances between each sample was at least 24 mm and the distances between the samples and the rim of the petri dish ranged from 10 to 15 mm. After 48 h of incubation at 30 ± 2 °C, images of the papers were taken and the extents of fungal colonization on the papers were determined.

## Results and discussion

### Spectroscopic characterizations of ZnO NPs, TMSC and ZnO/TMSC

**Figure 2:**
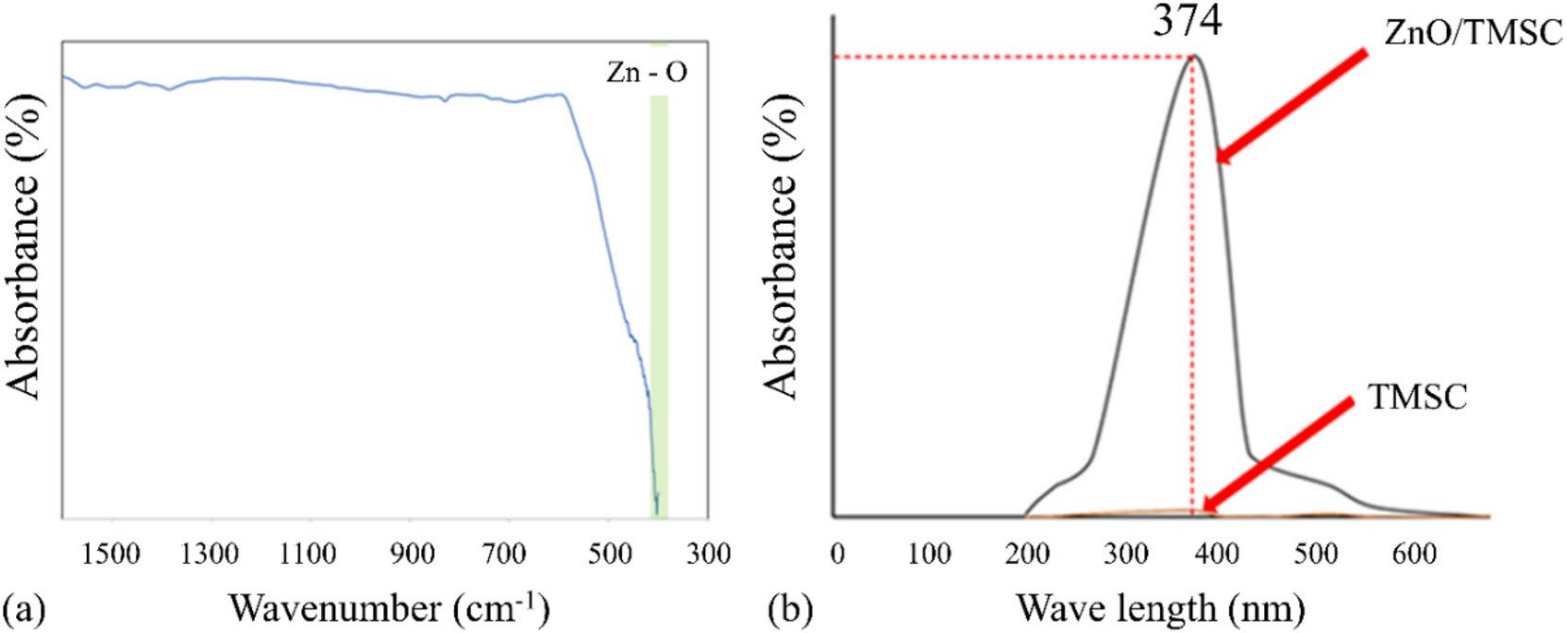
(**a**) ATR-IR spectrum of dry ZnO NPs and (**b**) UV–visible spectra of TMSC and 1% ZnO/TMSC.

Dry powder of ZnO NPs exhibited an absorption valley at wavenumbers between 402 and 440 cm^‒1^ (Fig. 2a) which corresponds to Zn–O stretching. The wavelengths at maximum absorption (*λ*_*max*_) of the TMSC solution and the ZnO/TMSC colloidal suspensions were determined in the UV–visible region and the absorption spectra are given in Fig. 2b. The *λ*_*max*_ of the ZnO/TMSC was 374 nm which is in the long-wave UV-A region whereas the TMSC merely exhibited tiny absorbances in the 300–400 nm region. The UV-A is not absorbed by the ozone layer and accounts for the majority of UV radiation reaching the Earth surface. In subsequent determinations of the UV protection efficacies of the coated papers, 365-nm UV light was employed due to its closeness to the *λ*_*max*_ of the ZnO/TMSC.

### Surface morphologies of coated papers after UV irradiations

Filter papers, with or without protective layers, were irradiated with 365-nm UV-A for 3 h. The conditions of coating were summarized as followed: (1) refers to uncoated paper; (2) refers to the paper coated with 2% w/v TMSC; (3), (4), and (5) refer to the papers coated with 2% w/v TMSC containing 0.1%, 0.5, and 1% ZnO NPs, respectively. For simplicity’s sake, the conditions (3), (4), and (5) were also referred to in the text as 0.1% ZnO/TMSC, 0.5% ZnO/TMSC, and 1% ZnO/TMSC coatings, respectively.

**Figure 3:**
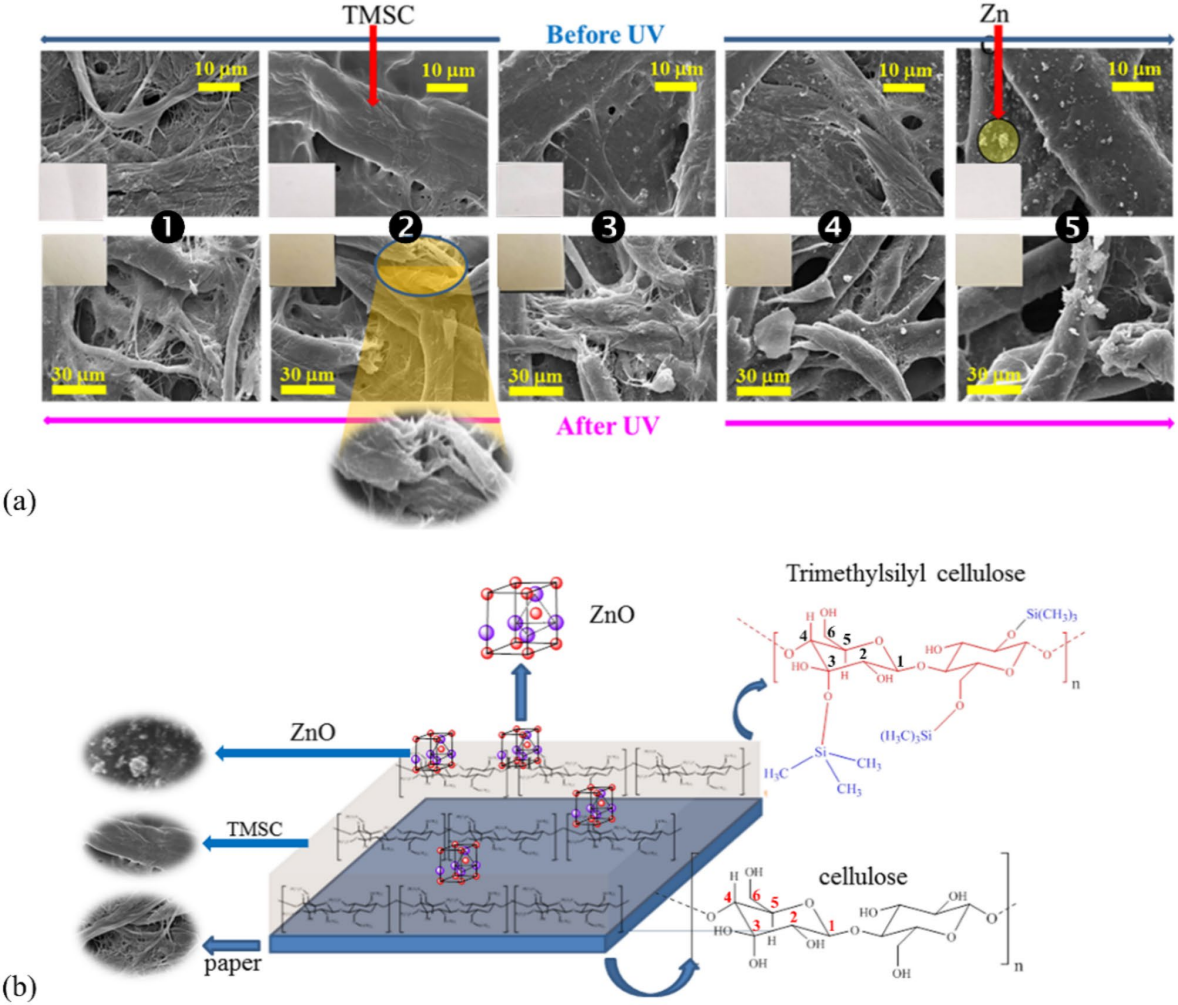
(**a**) SEM images of uncoated and coated papers before (top row) and after (bottom row) UV irradiations and (**b**) conceptual diagram depicting the structure of the top surface of the ZnO/TMSC-coated papers.

The SEM images of the textures of the papers without UV treatment are given in the top row of Fig. 3a. The surface of the uncoated paper was coarse with large, small, and very fine fibrils. Much smoother surface was observed in the TMSC-coated paper. The majority of TMSC chains aggregated to form large and smooth fibrils (Fig. 3a, top row, second column). Each glucose residue in celluloses possesses 3 hydroxyl (–OH) groups that can be converted to the trimethylsiloxyl (–O–Si(CH_3_)_3_; TMSO) groups. In TMSC with very high degree of substitution (2.8), relatively small numbers of hydroxyl groups were present in TMSC molecules and the numbers of hydrogen bonding between the TMSC molecules and the filter paper surfaces were limited as a result. Small numbers of intermolecular interactions between the coating polymer and the substrate promoted the aggregations of the TMSC chains.

In papers coated with ZnO/TMSC, protective films of ZnO-decorated TMSC matrices were formed on the surfaces. The conceptualized structure of the top surface of the ZnO/TMSC-coated papers is given in Fig. 3b in which the TMSC matrix was depicted as a thin flat layer with ZnO-decoration on top. ZnO particles of various sizes scattered all over the outer surfaces of TMSC fibrils. Owing to the hydrophilic nature of ZnO surfaces, it is arguable that only a small portion of ZnO NPs was trapped inside TMSC fibrils. As expected, higher concentrations of ZnO NPs led to higher densities of ZnO particles on the TMSC layers (Fig. 3a, top row, third to fifth columns). Even though the surfaces of TMSC fibrils were hydrophobic (discussed in more details below), the large surface areas of ZnO NPs allowed the particles to adhere to the TMSC fibrils. Colloidal precipitation was a simple method to apply ZnO NPs onto the surfaces of paper using TMSC as a binder. However, aggregation of ZnO NPs to form larger ZnO particles occurred in the present coating procedure. The optical and biological performances of ZnO were likely to be negatively affected as the aggregation reduced the total surface areas of ZnO and also led to lower particle density and uniformity of ZnO decoration.

With regard to UV-induced oxidations of celluloses, the textures of the UV-irradiated papers were destroyed to various extents (Fig. 3b, bottom row). This is the nature of cellulose and its derivatives that degrade on exposure to high-energy radiations. Since ZnO NPs were used as a UV filter in sunscreen formulations, it is expected that, with ZnO particles studded on the surfaces, the intensity of UV that reached the TMSC matrix and cellulose substrate was dramatically reduced at higher concentrations of ZnO. It is evident from the last column of Fig. 3b that the smoothness of TMSC surfaces on the 1% ZnO/TMSC-coated paper was preserved to a large extent. ZnO NPs at high concentrations were able to protect the coated paper from damages caused by UV exposure. To be accurate, ZnO NPs protected the underlying TMSC layers against UV-induced damages and the rate of deterioration of the cellulose substrate was also attenuated.

### Wettabilities of coated papers after UV irradiations

**Figure 4:**
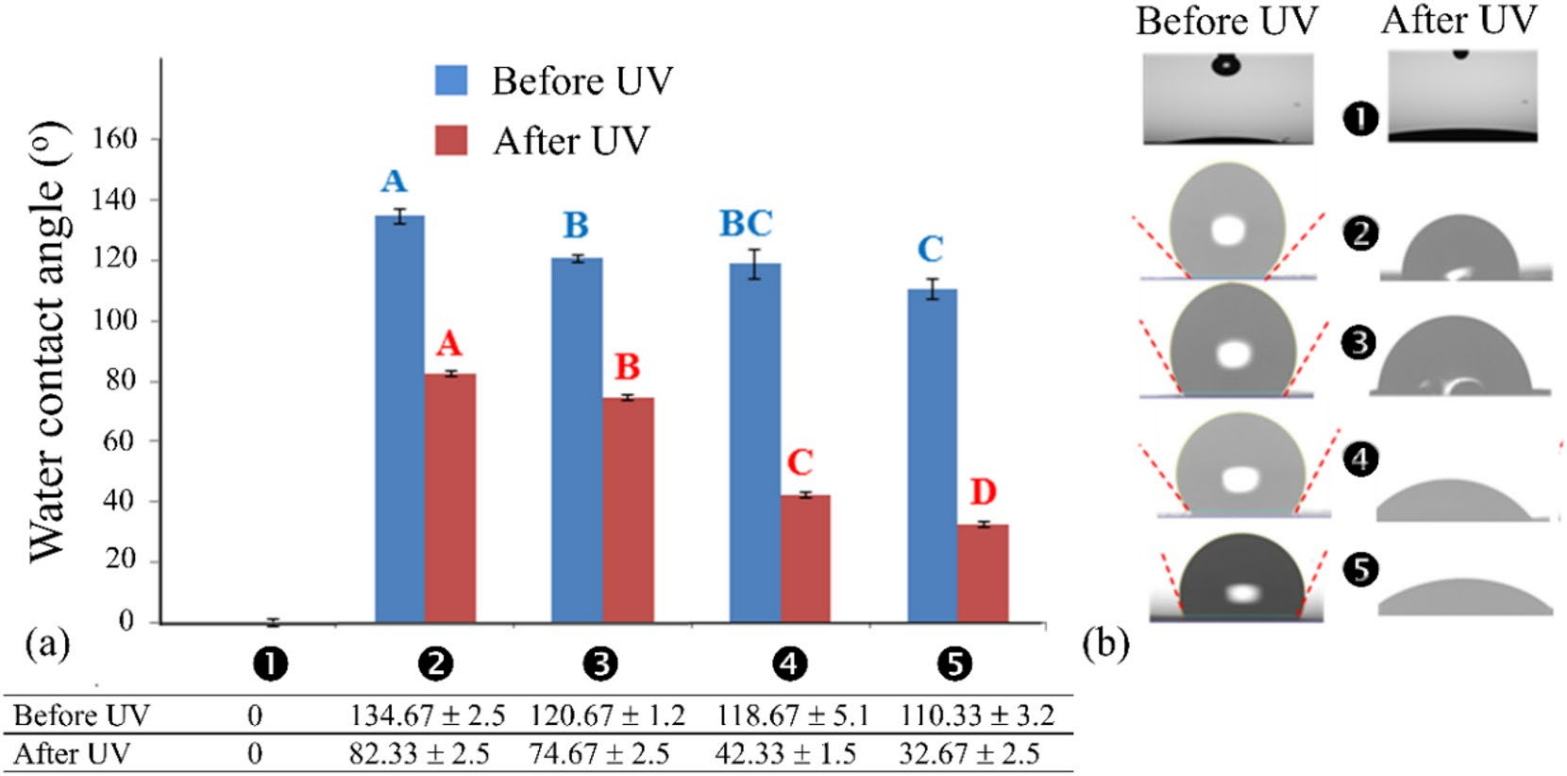
(**a**) Water contact angles before and after UV irradiation and (**b**) the appearances of water droplets on paper before (left column) and after (left column) UV irradiation.

The water contact angle values and the appearances of water droplets on the surfaces of the papers are given in Fig. 4. The contact angles of uncoated paper before and after UV irradiation were both zero due to the highly polar hydroxyl groups (on C2, C3, and C6 of glucose residues; Fig. 3b) exposed on the paper’s surface. When papers were coated with TMSC, with or without ZnO NPs, the coated papers’ surfaces became hydrophobic with water contact angles ranging from 110.33 to 134.67 (Fig. 4a). The hydrophobicity of TMSC or ZnO/TMSC film arose from trimethylsilyl (–Si(CH_3_)_3_; TMS) groups abundantly present on TMSC. Water cannot solvate the TMS groups exposed on the surface of TMSC fibrils.

**Figure 5:**
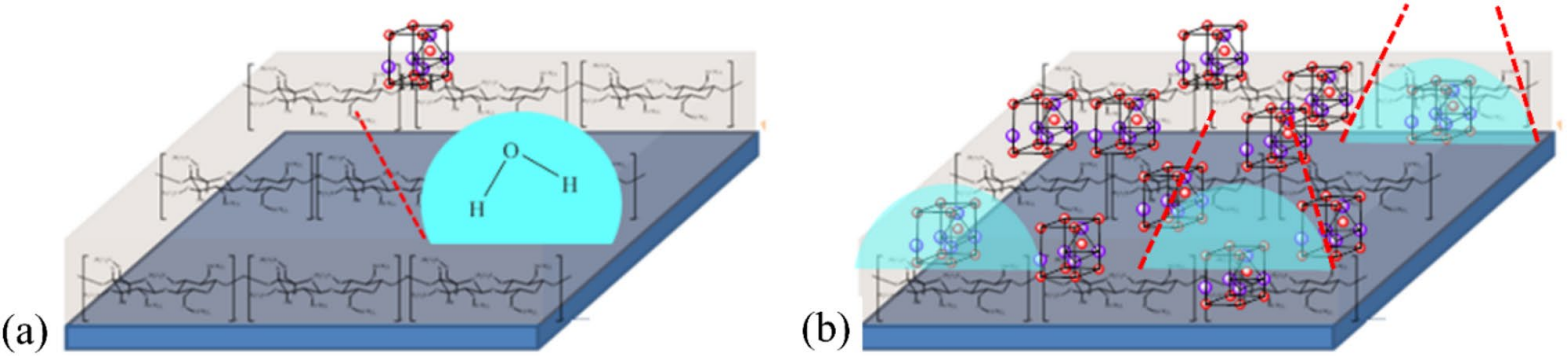
Shape and the corresponding contact angle of a water droplet on the surface of ZnO/TMSC film at (**a**) low and (**b**) high concentrations of ZnO.

Contact angles significantly decreased (*p* ≤ 0.05) when ZnO particles were present on the coated papers’ surfaces. Higher concentrations of ZnO resulted in smaller contact angles. The mean contact angle of 1% ZnO/TMSC-coated papers were significantly smaller than that of the 0.1% ZnO/TMSC-coated ones. With regard to the ZnO/TMSC-coated papers, positive correlations between ZnO concentrations and cosine values of the contact angles were observed with coefficients of determination (*R*^*2*^) of 0.9219 and 1.0000 for linear and quadratic relationships, respectively (data not shown). ZnO is a polar ionic compound. Through dipole–dipole interactions with water molecules, water readily adsorbed on ZnO particle surfaces. ZnO particles acted as hydrophilic anchoring points for water droplets. Higher densities of ZnO particles promoted the spreading of water droplets (Fig. 4b, left column). The effects of ZnO NPs concentrations on water contact angles of the ZnO/TMSC films were depicted graphically in Fig. 5a,b for low and high densities of ZnO particles, respectively.

UV irradiation led to significant reductions in water contact angles (*p* ≤ 0.05). The surfaces of UV-irradiated papers were considered hydrophilic owing to the contact angles that were less than 90°. After UV irradiations, larger drops in values of contact angle were observed at higher ZnO concentrations. The contact angles of coated papers were reduced by 38.8, 38.1, 64.3, and 70.4% for, TMSC-, 0.1% ZnO/TMSC-, 0.5% ZnO/TMSC-, and 1% ZnO/TMSC-coated papers, respectively. The most likely cause was because the surfaces of TMSC fibrils were less hydrophobic due to reduced degrees of trimethylsilyl (TMS) substitution. The hydrophobic TMS moieties on the surfaces of TMSC fibrils were partially removed on exposure to UV radiation (verified in ATR-IR spectra, discussed in more details below). As a result, ZnO particles on the surfaces were less hindered. Large-diameter TMSC fibrils made the surface uneven and a significant proportion of ZnO particles located deep on the grooves and trenches. Damages of TMSC caused by UV exposure evened out the surface and exposed more ZnO particles. It is also possible that, on exposure to UV radiation, the surfaces of ZnO particles were activated and the coating layers became more hydrophilic as a result. The UV-induced superhydrophilicity of ZnO thin film was reported^30^.

The TMSC protective layers acted as the moisture barrier that reduced the amounts of ambient moistures absorbed by the coated papers. Thus, the susceptibilities of coated papers to quality-degraded processes induced or promoted by high moisture content (such as fungal spoilage) were also decreased as a result. Even though the decoration of the TMSC matrix with ZnO particles resulted in lower water-repelling abilities, the surfaces of UV-irradiated ZnO/TMSC-coated papers were more hydrophobic than those of nonirradiated uncoated papers.

### Yellowing of celluloses after UV irradiations

**Figure 6:**
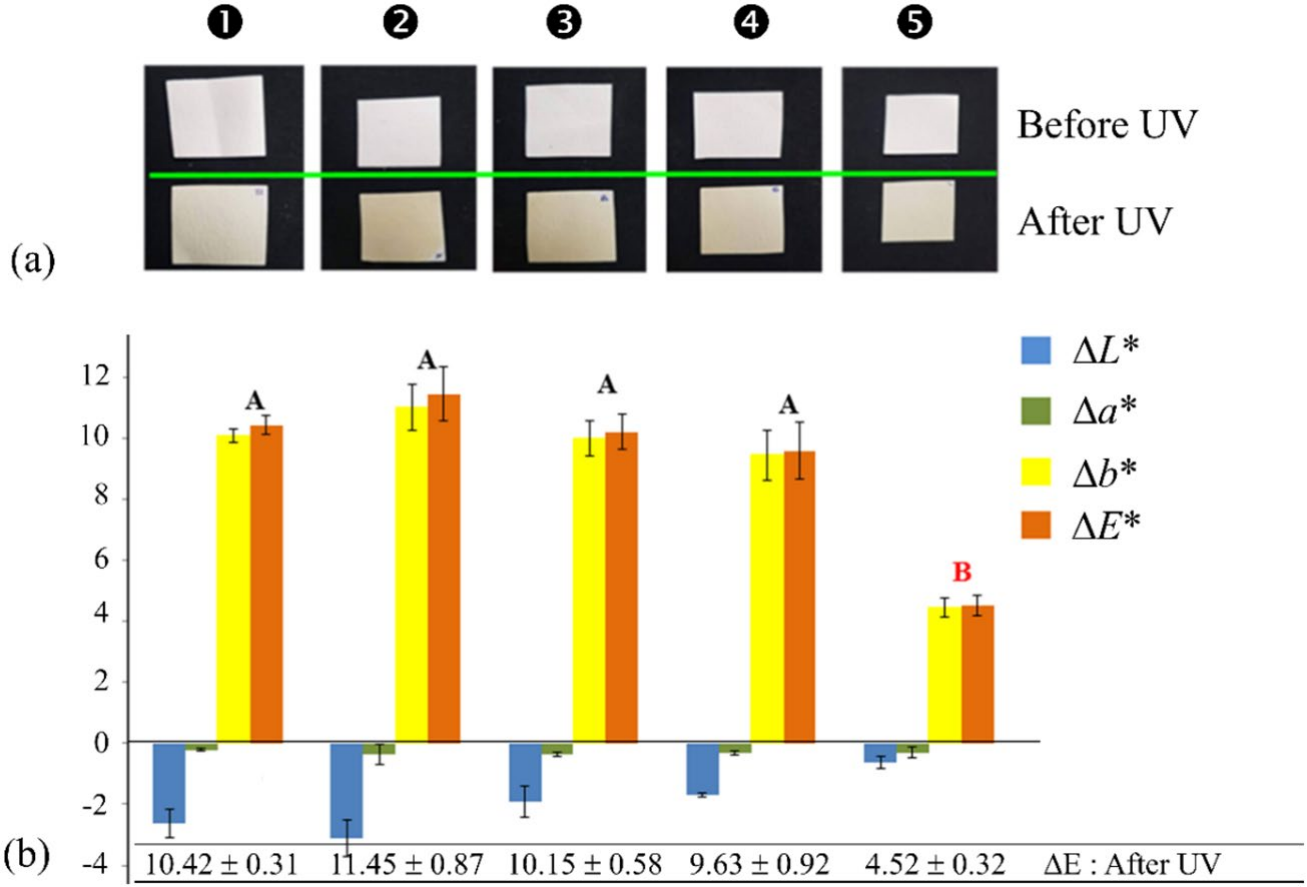
(**a**) Photograph images of filter papers before and after UV irradiations and (**b**) L*, a*, b* color coordinates along with color change (ΔE*) values after UV irradiations.

With naked eyes, there was no noticeable difference between the coated and uncoated papers before UV irradiation. All papers were plain white (Fig. 6a, top row) and turned yellow after UV irradiation (Fig. 6a, bottom row). The L*, a*, b* color coordinates of nonirradiated and UV-irradiated papers along with the extents of color changing (ΔE*) were plotted in Fig. 6b.

As assessed by ΔE*, the extent of yellowing of TMSC-coated papers due photo-oxidation was greater than that of the uncoated paper. This may be because TMSC is a cellulose derivative. In this regard, TMSC absorbed UV radiation and promoted self-damages. Therefore, without any UV filter incorporated into the TMSC matrix, further yellowing occurred. Lower ΔE* values were observed in papers coated with ZnO/TMSC. This is because ZnO is able to absorb and filter out UV radiation^31^. Papers coated with 0.1% and 0.5% ZnO/TMSC exhibited a non-significant decrease in ΔE* compared to those of the uncoated and TMSC-coated papers (*p* > 0.05). This may be because, at such concentrations, the densities of ZnO particles were too low to filter out a significant proportion of UV radiation. The intensities of residual UV were able to oxidize the TMSC and cellulose chains to a great extent so the ΔE* values remained high. With 1% ZnO/TMSC protective layers, the ΔE* value was the lowest and significantly different from those of the other coated papers (*p* ≤ 0.05). The density of ZnO particles was high enough to filter UV radiation to a sufficiently low intensity that led to diminutions of yellowing. Therefore, at high concentrations of ZnO NPs, damages induced by UV in the TMSC matrices were lessened. With 1% ZnO/TMSC coating, the rate of paper yellowing was slowed down to an appreciable degree. Slower rates of yellowing can be achieved with ZnO/TMSC coating at higher concentrations of ZnO NPs. However, higher ZnO negatively affected the anti-fungal activities of the coated paper as discussed in below.

The effects of ZnO on the slowing down of wood browning caused by UV exposure were reported^32^. ZnO could reduce the direct impact of UV radiation on papers via UV scattering and UV absorption. Partial inhibition of the depolymerization of cellulose and its derivatives into smaller organic molecules reduced the accumulation of the compounds responsible for the undesirable yellowish or brownish hue.

The yellow colors of celluloses were also reported to arise from the oxidations of hydroxy (–OH) groups on the carbon ring of glucose residues. The oxidation converts alcohol functional groups to carbonyl (〉C=O) groups in which aldehyde (–CHO) and keto (–CO–) functional groups are possible. The aldehyde functional groups can be further oxidized to carboxyl (–COOH–) groups. The aldehyde and keto groups act as the chromophores responsible for the yellow colors of aged papers. In the absence of aldehyde/keto groups on the cellulose chains, the carboxylic groups cannot act on their own as chromophores. In oxidized celluloses, all these functional groups are present and the carboxylic groups intensify the yellow color^33^.

### Tensile strengthening and strength retention after UV irradiation

**Figure 7:**
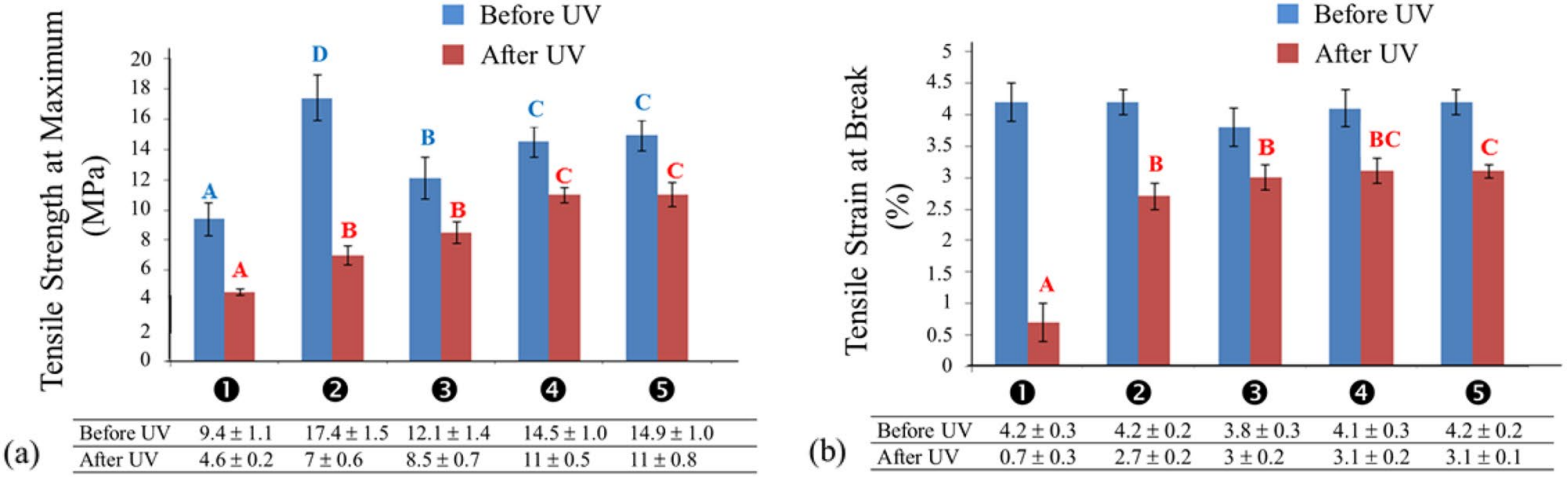
(**a**) Tensile strength at maximum and (**b**) tensile strain at break of papers before and after UV irradiation.

TMSC coating increases the strength of the coated paper^26^. The tensile strengths at maximum given in Fig. 7a showed that papers coated with TMSC were stronger. As expected, the UV-irradiated TMSC-coated paper was stronger than the irradiated uncoated one. It is worth to mention that, after UV irradiations, the tensile strength of the uncoated paper reduced to ~ 49% of the nonirradiated paper. In TMSC-coated paper, the tensile strength reduced to ~ 40% after UV irradiation. Without ZnO, the TMSC film was more susceptible to UV-induced damages than the uncoated paper. This observation agreed well with the above-mentioned higher ΔE* value of the TMSC-coated compared to that of the uncoated one.

With 0.1% ZnO/TMSC coating, the tensile strength of the coated paper was significantly lower (*p* ≤ 0.05). Significant increases in tensile strengths were observed with 0.5 and 1% ZnO. It is unclear why a low concentration of ZnO NPs led to a dramatic reduction in tensile strength but the strengths increased at high ZnO NPs concentrations rather than further decreased. Since higher ZnO concentrations resulted in less hydrophobic surface, paper coated with high ZnO concentrations absorbed more moistures and greater degrees of reductions in tensile strengths were expected. One possible explanation is that high ZnO concentrations promoted the formation of TMSC fibrils of larger diameters. With equal total cross-sectional areas, one large fibril is stronger than 2 smaller fibrils combined.

Considering the upward trend of tensile strength of the ZnO/TMSC-coated papers, tensile strength comparable to that of the TMSC-coated paper was not possible to achieved if ZnO NPs were incorporated into the TMSC matrix. Therefore, ZnO NPs were detrimental to the strength of coated papers without UV treatment. However, after UV irradiation, the tensile strengths of 0.5% and 1% ZnO/TMSC-coated papers were higher than that of the uncoated and nonirradiated paper. This observation confirmed that ZnO helped lessen the UV-induced damages in the TMSC matrix.

With regard to the stretchable lengths of the nonirradiated papers, there was no significant difference in the tensile strain at break between uncoated and coated (with or without ZnO) papers (Fig. 7b). This indicates that the TMSC coating layers were harder to stretch than the cellulose substrate but their elasticities were comparable. The conversion of hydroxyl groups to trimethylsiloxyl groups possibly alter the way TMSC chains aggregate to form fibrils but does not affect the elasticity of the fibrils.

After UV irradiation, the tensile strain of papers coated with TMSC was significantly higher than that of uncoated paper (*p* ≤ 0.05). Because UV partially destroyed the paper’s cellulose structure, uncoated papers exhibited a very low tensile stain. However, with TMSC coating, UV-induced damages first occurred at the TMSC protective film. Therefore, the damages in the underlying cellulose substrate were not as severe and extensive as those on the unprotected paper. The TMSC-coated paper retained much of its tensile strain. Since ZnO filtered out a portion of UV, damages in the ZnO/TMSC film and cellulose substrate were smaller than those occurred in the TMSC-coated paper. The tensile strain of 1% ZnO/TMSC-coated paper was the highest and amounted to 442.85% and 114.81% of the strains of uncoated and TMSC-coated papers, respectively.

### Spectroscopic investigations of UV-induced damages and TMSC surface alteration

**Figure 8:**
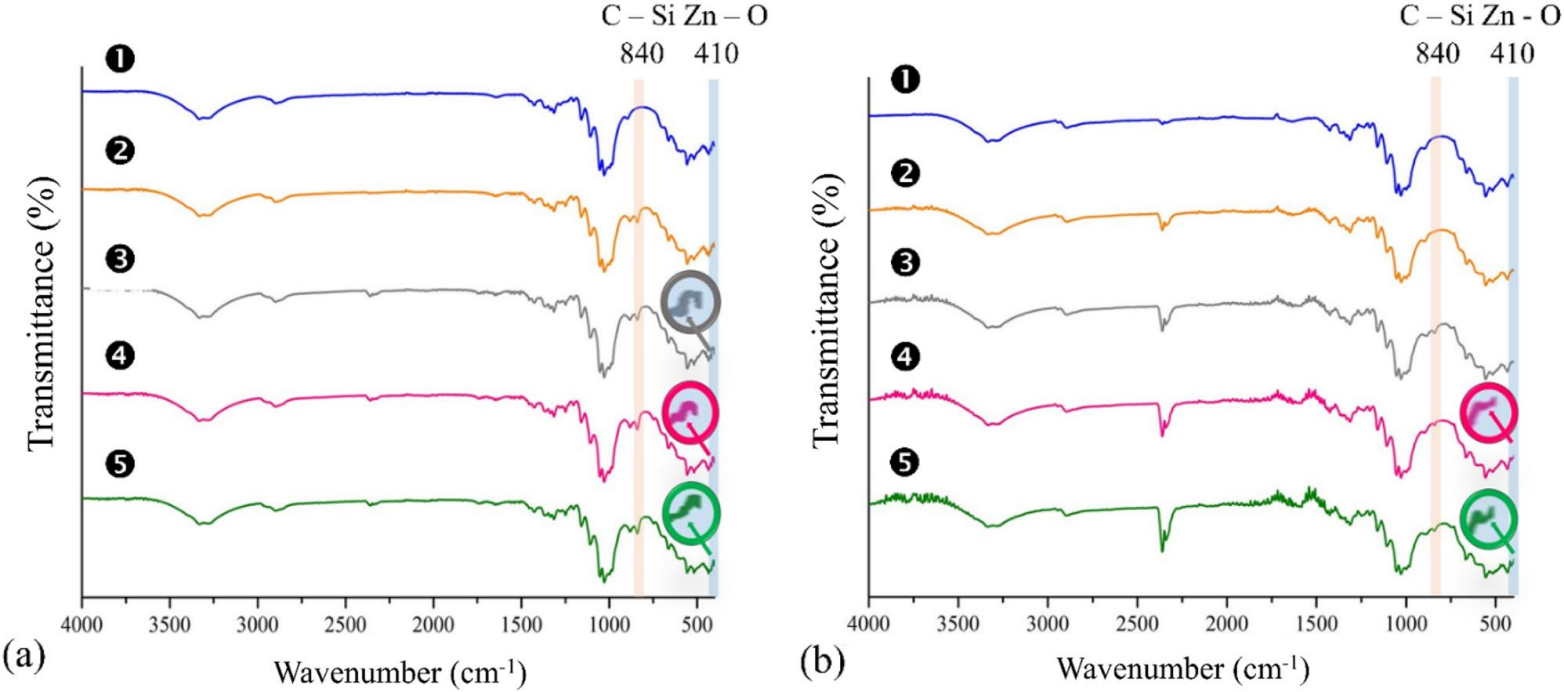
ATR-IR spectra of papers before (**a**) and after (**b**) UV irradiations.

The ATR-IR peak at the wavenumber 840 cm^‒1^ corresponds to C–Si bond. All of the ATR-IR spectra of nonirradiated papers (Fig. 8a) had a peak at the wavenumber 840 cm^‒1^ except for the uncoated one. Therefore, the presence of TMSC on TMSC- and ZnO/TMSC-coated papers and the absence of TMSC on uncoated paper were verified. With regard to the presence or absence of ZnO, Zn–O stretching resolved at 403.04–433.4 cm^‒1^. The uncoated and TMSC-coated papers had no peak at this position whereas the Zn–O stretching peaks appeared in the IR spectra of the papers coated with ZnO/TMSC.

The IR spectra of the irradiated papers are given in Fig. 8b. With regard to the Zn–O peaks, there was no difference between the peaks of nonirradiated and UV-irradiated papers with the same coating, indicating that ZnO was still present after UV irradiations. On the other hand, peak at wavenumber 840 cm^‒1^ of TMSC-coated paper was disappear after irradiation. This suggested that the majority, if not all, of the TMS groups were removed from the paper surface. After oxidation, TMSC produces H^+^ capable of reducing the trimethylsiloxyl (TMSO) groups to hydroxyl groups. The TMS groups were converted to gaseous trimethylsilanes. If all of the TMS groups were removed, unsubstituted cellulose was regenerated from TMSC. In other words, the TMS moieties acts as the sacrificing H^+^ scavenger. This process reduces the destruction of paper coated with TMSC, which is consistent with the research of Amonkitbamrung et al. (2015).

All ZnO/TMSC-coated papers showed the C–Si peak, indicating that the removals of the TMS groups were far from complete and large amounts of TMS groups remained present on the paper. This observation suggests that smaller amounts of H^+^ were released when the ZnO/TMSC-coated papers were exposed to UV. Smaller amounts of H^+^ mean smaller extents of oxidations of TMSC and cellulose occurred in the presence of ZnO particles. The presences of C–Si peaks in IR spectra of ZnO/TMSC-coated payers provide an indirect evidence that ZnO helped protect the TMSC matrix and the underlying celluloses by reducing the extents of UV-induced oxidations.

**Figure 9:**
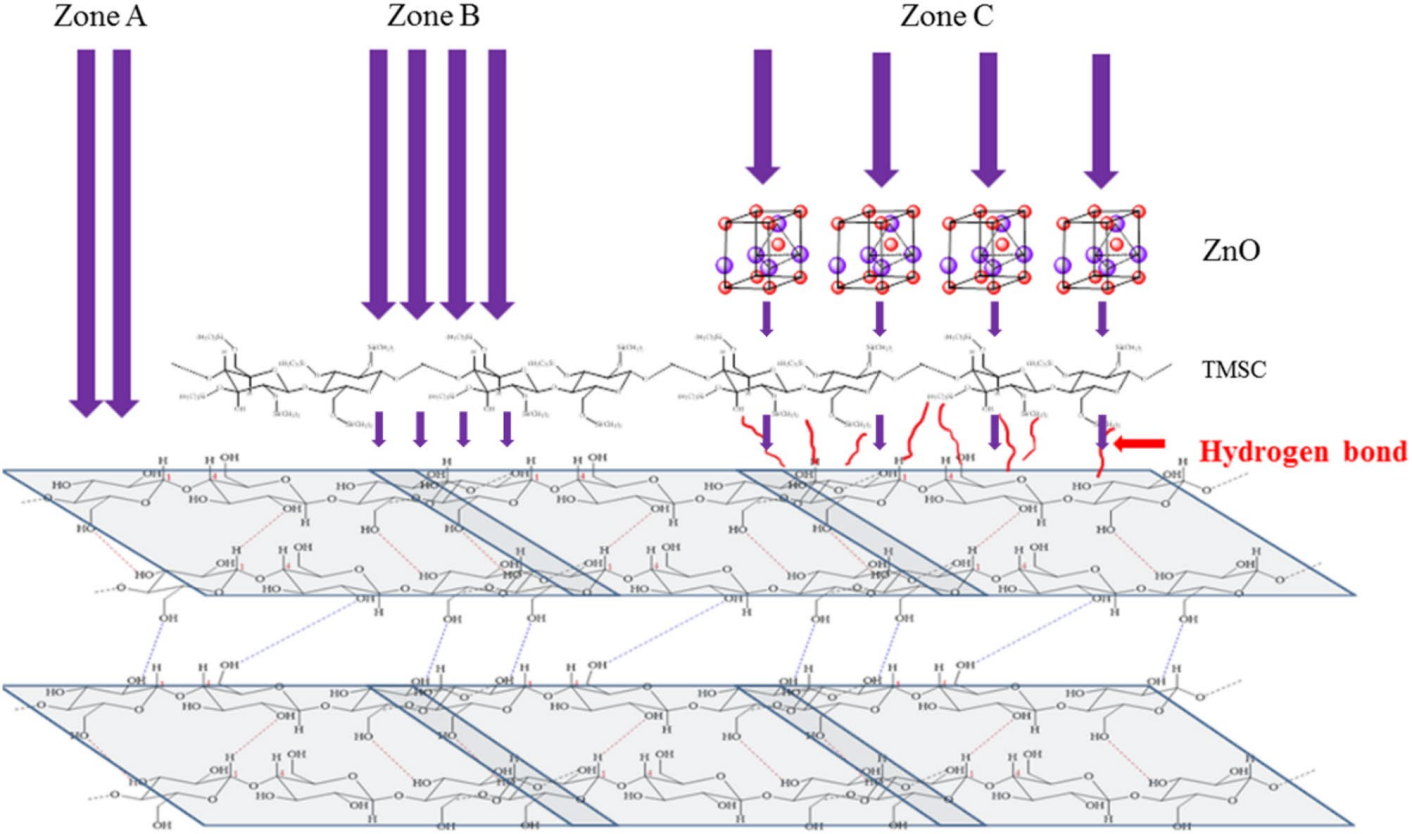
Top surfaces of papers without (Zone A) and with TMSC- (Zone B) or ZnO/TMSC (Zone C) protective layer. The purple arrows represent UV radiation. Thicker arrow indicates higher UV intensity.

The irradiation with high-power 365-nm UV for 3 h was employed to introduce accelerated UV-induced damages to the coated paper. Figure 9 summarizes how paper surfaces with different coating layers were damaged by UV irradiation. Zone A is exposed directly to UV radiation. Photo-oxidation of cellulose releases H^+^ that causes further damages to cellulose structure. Zone A is highly hydrophilic and very fragile. The tensile strength and strain dramatically drop after UV irradiation.

Zone B is protected and strengthened mechanically by TMSC layers and is highly hydrophobic before UV irradiation. The TMS groups of TMSC reacts with the released H^+^ and reduces the extents of further damages. The majority of the TMS groups is removed and the irradiated surface of Zone B becomes hydrophilic. The destruction on the TMSC is high while that occurred on the cellulose substrate is much smaller. The UV radiation may destroy the hydrogen bonds between the TMSC and cellulose substrate. Both of the tensile strength and strain are much higher than those of Zone A.

Zone C is protected by the ZnO/TMSC layer. Before irradiation, Zone C is not as strong and hydrophobic as Zone B. At high ZnO concentration, the UV intensity reaching the TMSC matrix is significantly reduced. A smaller amount of H^+^ is released and a significant proportion of the TMS groups is retained on the surface but the irradiated surface of Zone C is hydrophilic due to the presence of ZnO particles. The surface of Zone C is yet much less hydrophilic compared to that of Zone A due to the presence of the TMS moieties. Damages in the TMSC matrix is smaller than those present in Zone B. The cellulose substrate of Zone C is minimally destroyed or remains intact. Both of the tensile strength and strain are the highest.

### Anti-fungal activities

**Figure 10:**
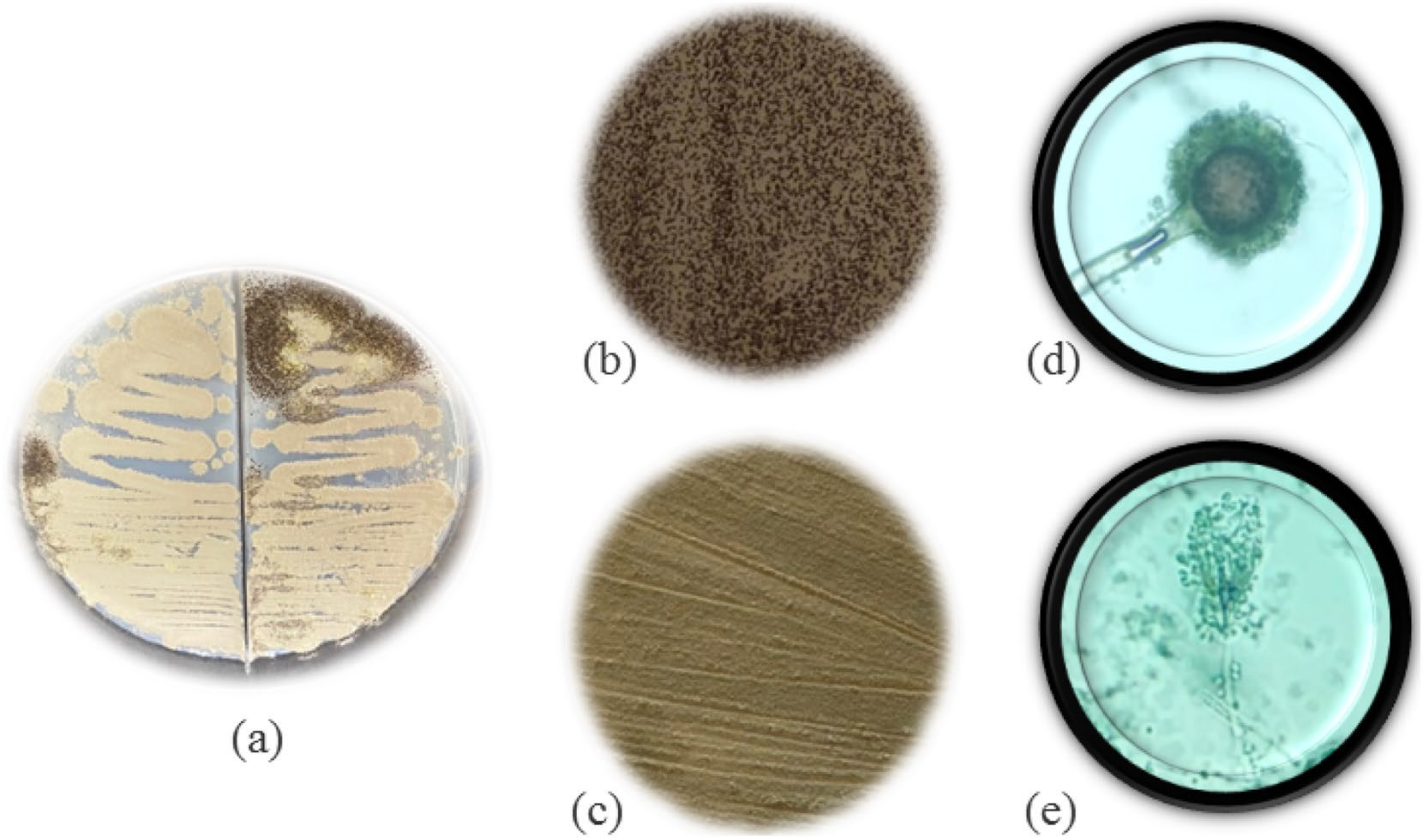
(**a**) Molds obtained surface swab of a book cover; morphology of *Aspergillus* sp.: (**b**) colony texture and (**d**) spores and conidiophores; morphology of *Penicillium* sp.: (**c**) colony texture and (**e**) spores and conidiophores. (**d**,**e**) are at 40 × magnification.

From the surface swab of a book cover, two species of rapidly growing mold were found on PDA culture plates (Fig. 10a). The mold species were identified based on colony textures and the morphologies of their reproductive tissues. The 2 fungi were *Aspergillus* sp. with dark-brown colonies (Fig. 10b) and *Penicillium* sp. with brownish yellow colonies (Fig. 10c). Both species are *Ascomycetes* which produce asexual spores called conidia. The spore-bearing hyphae are called conidiophores. The conidia of the former were black and produced on globular conidiophores (Fig. 10d) whereas the latter’s conidiophores produced branches of spore-chains that arrange into dense brush-like structures (Fig. 10e).

It was reported in the literatures that molds, especially those in the genera *Aspergillus* and *Penicillium*, are the most common fungi responsible for fungal spoilage of papers kept in bad conditions^34^. Improving the conditions of paper/book storage can minimized the growths of opportunistic fungi^35^. For valuable pieces of writing on cellulose pulp, elimination of potential sources of fungal contamination is required. Anti-fungal coating is a promising method that fortifies the protection level.

Anti-fungal activities of papers with 1% ZnO/TMSC protective films were investigated by placing the paper on top of inoculum of either *Aspergillus* sp. or *Penicillium* sp. evenly spread on PDA plates. The plates were covered and incubated at optimal growth temperature for 2 days. In this way, the papers were placed in a humid chamber and in direct contact with the molds. The efficacies of growth inhibitions were evaluated qualitatively according to the proportion of paper top surface that cover with the molds.

**Figure 11:**
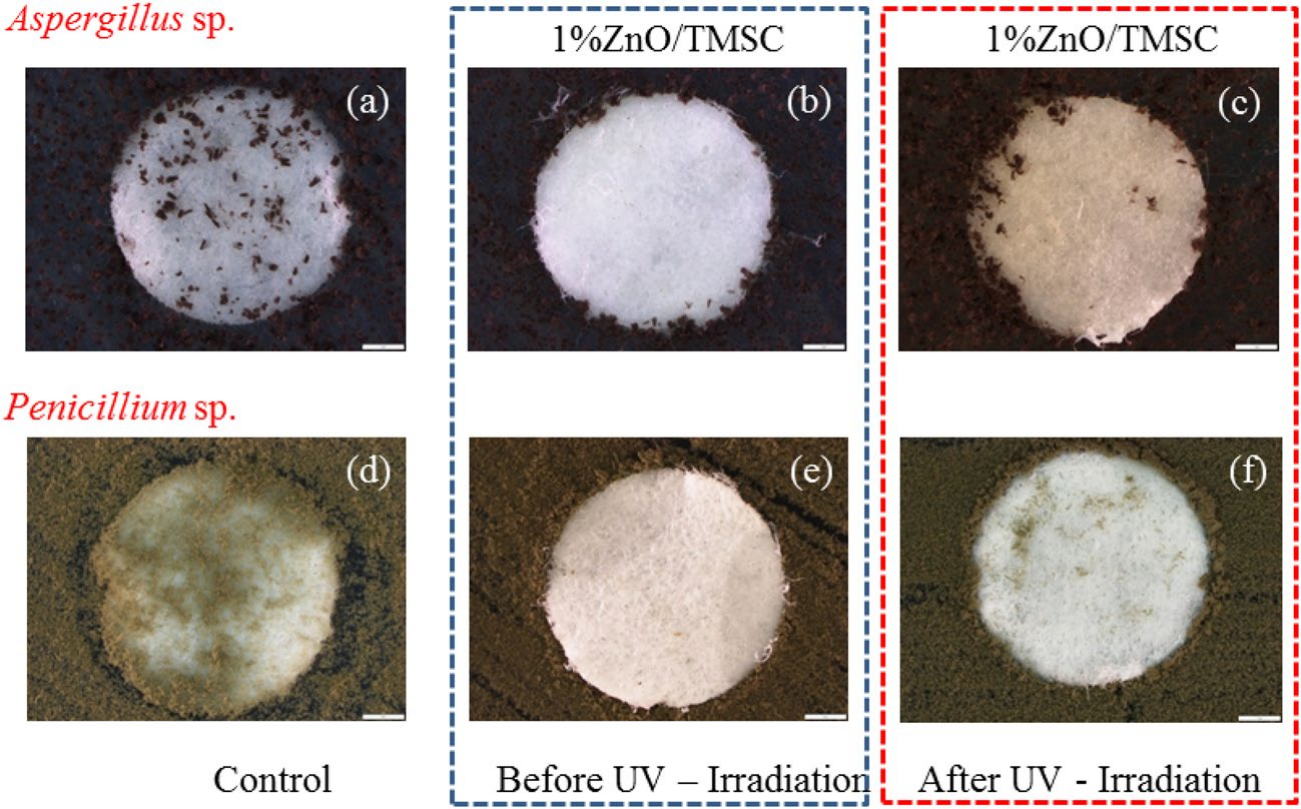
Growths of *Aspergillus* sp. (**a**–**c**) and *Penicillium* sp. (**d**–**f**) on nonirradiated uncoated papers (**a**,**d**) or on 1% ZnO/TMSC-coating papers before (**b**,**e**) and after (**c**,**f**) UV irradiations.

On an uncoated paper without UV treatment, *Penicillium* sp. was found throughout the paper top surface. More than 3 quarters of the paper top surface (84.36%) were covered with mycelia of *Penicillium* sp. (Fig. 11d). Even though smaller growth of the fungi (23.36% of the paper top surface) was found on the uncoated papers, *Aspergillus* sp. was observed at the center (Fig. 11a). With 1% ZnO/TMSC coating, the fungi grown of the peripheries of the plates began to invade paper surface. Only a smalls number of visible colonies of *Penicillium* sp. were observed on the edge of the coated paper (Fig. 11e). Many large dark-brown patches of *Aspergillus* sp. were observed at the outermost area of the coated papers. More than 90% of the coated paper surface was free of *Aspergillus* sp. (Fig. 11b). This indicates that ZnO with anti-fungal properties acted in concert with the hydrophobic nature of the coated paper to suppress the invasions of the fungi.

In term of fungal growth inhibition, it is evident from Fig. 11 that UV irradiation made the 1% ZnO/TMSC protective layers much weaker. *Penicillium* sp. were observed at the center of paper (Fig. 11f) whereas *Aspergillus* sp. invasion progressed much further to the center (Fig. 11c). The areas covered with the *Penicillium* sp. were increased from 12.49% on the coated paper to 17.74% after UV irradiation. The areas covered with the *Aspergillus* sp. were increased from 16.20% on the coated paper to 23.16% after UV irradiation.

The losses in the efficacies of fungal growth inhibition after UV irradiation were due to the increase in surface hydrophilicity and the partial loss of ZnO particle from the protective layer. ZnO/TMSC protective layers can inhibit fungal growth in 2 ways. The coating layers inhibited (1) the growth of the fungal hyphae and (2) the germinations of the fungal spores. In this regard, the ability to inhibit spore germination of the irradiated ZnO/TMSC layers seem to be severely impaired by UV irradiation.

**Figure 12:**
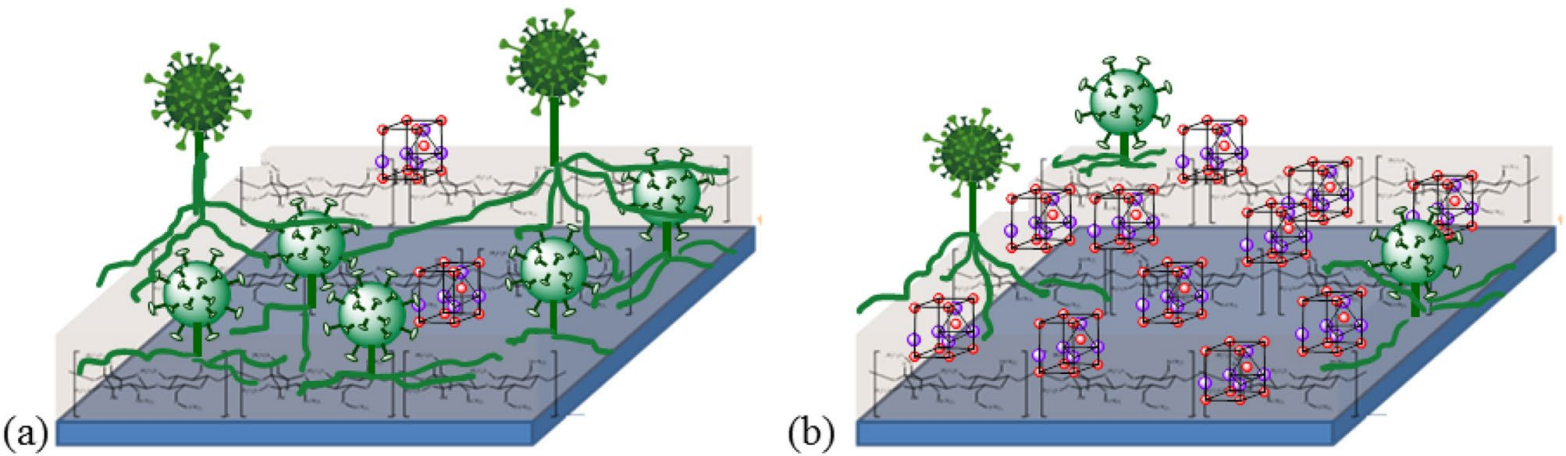
Fungal growth on papers coated with ZnO/TMSC at (**a**) low and (**b**) high ZnO concentrations.

Even with UV-induced damages, the anti-fungal performance of the irradiated coated papers was still much better than that of the nonirradiated uncoated paper. However, complete growth inhibitions of the molds were not able to achieved with 1% w/v ZnO NPs. The ZnO NPs concentration was too small compared to those used in the study by SE and HE^36^. Efficient bacterial controls on cellulose-based ZnO nano-composite films were also reported at much higher ZnO concentrations^37^. As depicted in Fig. 12, higher densities of ZnO particles may improve the fungal resistance of the coated papers^17^, but excessive amounts of ZnO are very likely to greatly increase the hydrophilicity of the surfaces of coated papers. It counteracts the actions of ZnO and impairs the paper’s anti-fungal performance as it promotes higher water availability that better supports fungal growth. The ZnO at 1% w/v may be a good compromise that does not make the surface of the coated paper too hydrophilic and can still suppresses the growth of molds to an appreciable extent.

### Implications and potential applications

Overall results presented in this work demonstrated that 1% ZnO/TMSC-protected papers can be used as a medium for art and image/photo reproduction with preserving the age and quality of recorded data and knowledges and written artistic works. Specifically, they have a high potential for the future application as robust and durable paper-based packages for foods and portable electronic devices.

## Conclusion

This study aimed to increase the durability and reduce the rate of photo-oxidation of papers. Oxidations of celluloses cause white papers to progressively change their colors to undesirable dark-yellow hues. To this end, the thin protective ZnO/TMSC layers were applied on papers using the colloidal precipitation method. The strains at break of papers were not affected by ZnO/TMSC coating. The TMSC layers helped fortify the papers by increasing their tensile strengths at maximum and were also responsible for the coated papers’ high surface hydrophobicities. On the other hand, ZnO NPs rendered the ZnO/TMSC-coated papers slightly weaker and less hydrophobic than the TMSC-coated ones. As a UV filter, ZnO NPs reduced the UV intensity reaching the TMSC layer and the cellulose substrate and, therefore, damages caused by UV-induced oxidations were lessened as a result. Coating with 1% ZnO/TMSC was effective in the suppressions of *Aspergillus* sp. and *Penicillium* sp. colonizations on the paper. Even though the water contact angle of the 1% ZnO/TMSC-coated paper was dramatically dropped after UV-irradiation, the 1% ZnO/TMSC-coated paper was much more superior to the nonirradiated uncoated paper in terms of anti-fungal properties. Concerning the yellowing of the coated papers, a significant reduction in color change (ΔE*) was observed in papers coated with 1% ZnO/TMSC. With the present coating system, smaller ΔE* values could be achieved with higher concentrations of ZnO NPs. However, a better anti-UV performance does not guarantee a better anti-fungal performance. Even though the ZnO NPs exhibit anti-microbial properties, it is very likely that higher amounts of ZnO NPs also lead to more hydrophilic surfaces.

## Data Availability

All data generated or analyzed during this study are included in this published article.

## Abbreviations

DDM: Dish diffusion method
eV: Electron volt
ATR-IR: Attenuated total reflection infrared
HMDSO: Hexamethyl dixiloxane
MeV: Mega electron volt
MPa: Mega Pascal
PDA: Potato dextrose agar
pen/str: Penicillin/streptomycin
TMSC: Trimethylsilyl cellulose
UV: Ultraviolet
UV-spectroscopy: Ultraviolet spectroscopy
ZnO: Zinc oxide
ZnO/TMSC: Zinc oxide/trimethylsilyl cellulose

## References

[1] Kumar V, Khan A, Rabnawaz M (2023). A plant oil-based eco-friendly approach for paper coatings and their packaging applications. Prog. Org. Coat..

[2] Area MC, Cheradame H (2011). Paper aging and degradation: Recent findings and research methods. Bioresources.

[3] Bernerd F, Passeron T, Castiel I, Marionnet C (2022). The damaging effects of long UVA (UVA1) rays: A major challenge to preserve skin health and integrity. Int. J. Mol. Sci..

[4] Geffertova J, Geffert A, Deliiski N (2016). The Effect of light on the changes of white office paper. Key Eng. Mater..

[5] Marsh K, Bugusu B (2007). Food packaging: Roles, materials, and environmental issues. J. Food Sci..

[6] Baty JW, Maitland CL, Minter W, Hubbe MA, Jordan-Mowery SK (2010). Deacidification for the conservation and preservation of paper-based works: A review. Bioresources.

[7] Hon NS (1975). Formation of free radicals in photo irradiated cellulose. I. Effect of wavelength. J. Polym. Sci Polym. Chem. Ed..

[8] Kolar J, Strlic M, Pentzien S, Kautek W (2000). Near-UV, visible and IR pulsed laser light interaction with cellulose. Appl. Phys. A.

[9] Maundrill ZC, Dams B, Ansell M, Henk D, Ezugwu EK, Harney M, Stewart J, Ball RJ (2023). Moisture and fungal degradation in fibrous plaster. Constr. Build. Mater..

[10] Bangar SP, Esua OJ, Nickhil C, Whiteside WS (2023). Microcrystalline cellulose for active food packaging applications: A review. Food Packag. Shelf Life.

[11] Hurra J, Heinzow B, Aurbach U, Bergmann KC, Bufe A, Buzina W, Cornely OA, Engelhart S, Fischer G, Gabrio T, Heinz W, Herr CEW, Kleine-Tebbe J, Klimek L, Köberle M, Lichtnecker H, Lob-Corzilius T, Merget R, Mülleneisen N, Nowak D, Wiesmüller GA (2017). Medical diagnostics for indoor mold exposure. Int. J. Hyg. Environ. Health.

[12] Jitianu M, Goia DV (2007). Zinc oxide colloids with controlled size, shape, and structure. J. Colloid Interface Sci..

[13] Lupan O, Chow L, Chai G, Schulte A, Park S, Lopatiuk-Tirpak O, Chernyak L, Heinrich H (2008). Biopolymer-assisted self-assembly of ZnO nanoarchitectures from nanorods. Superlattices Microstruct..

[14] Yiamsawas D, Boonpavanitchakul K, Kangwansupamonkon W (2009). Preparation of ZnO nanostructures by solvothermal method. J. Microsc. Soc. Thail..

[15] Wang RH, And JHX, Tao XM (2005). UV-blocking property of dumbbell-shaped ZnO crystallites on cotton fabrics. Inorg. Chem..

[16] Li C, Xie Y, Liu Q, Zheng Y, Zhang X, Dong W (2014). The formation and UV-blocking property of flower-like ZnO nanorod on electrospun natural cotton cellulose nanofibers. Fibers Polym..

[17] Sharma D, Rajput J, Kaith BS, Kaur M, Sharma S (2010). Synthesis of ZnO nanoparticles and study of their antibacterial and antifungal properties. Thin Solid Films.

[18] Zhang L, Jiang Y, Ding Y, Povey M, York D (2007). Investigation into the antibacterial behaviour of suspensions of ZnO nanoparticles (ZnO nanofluids). J. Nanopart. Res..

[19] Kairyte K, Kadys A, Luksiene Z (2013). Antibacterial and antifungal activity of photoactivated ZnO nanoparticles in suspension. J. Photochem. Photobiol. B.

[20] Seki M, Sonoda N, Morita T, Okayama T (2005). A new technique for strengthening book papers using cellulose derivatives. Restaurator.

[21] Seki M, Sonoda N, Hidaka S, Morita T, Okayama T (2010). A new technique for strengthening book papers with cellulose derivatives Part 2: Effect of cellulose derivatives on different types of paper. Restaurator.

[22] Sundholm F, Tahvanainen M (2003). Paper conservation using aqueous solutions of calcium hydroxide/methyl cellulose: 1. Preparation of the solution. Restaurator..

[23] Sundholm F, Tahvanainen M (2003). Paper conservation using aqueous solutions of calcium hydroxide/methyl cellulose 2. The influence of accelerated ageing temperature on properties of treated paper. Restaurator.

[24] Sundholm F, Tahvanainen M (2004). Paper conservation using aqueous solutions of calcium hydroxide/methyl cellulose. 3. The influence on the degradation of papers. Restaurator.

[25] Amornkitbamrung L, Mohan T, Hribernik S, Reichel V, Faivre D, Gregorova A, Engel P, Kargl R, Ribitsch V (2015). Polysaccharide stabilized nanoparticles for deacidification and strengthening of paper. RSC Adv..

[26] Amornkitbamrung L, Marnul MC, Palani T, Hribernik S, Kovalcik A, Kargl R, Stana-Kleinschek K, Mohan T (2018). Strengthening of paper by treatment with a suspension of alkaline nanoparticles stabilized by trimethylsilyl cellulose. Nano-Struct. Nano-Objects.

[27] Dang DS, Buhler JF, Stafford CD, Taylor MJ, Shippen JE, Dai X, England EM, Matarneh SK (2021). Nix Pro 2 and Color Muse as potential colorimeters for evaluating color in foods. LWT.

[28] Fakin D, Veronovski N, Ojstršek A, Božič M (2012). Synthesis of TiO_2_–SiO_2_ colloid and its performance in reactive dyeing of cotton fabrics. Carbohydr. Polym..

[29] Fakin D, Kleinschek KS, Kurečič M, Ojstršek A (2014). Effects of nanoTiO_2_–SiO_2_ on the hydrophilicity/dyeability of polyester fabric and photostability of disperse dyes under UV irradiation. Surf. Coat. Technol..

[30] Barreca D, Gasparotto A, Maccato C, Tondello E, Štangar UL, Patil SR (2009). Photoinduced superhydrophilicity and photocatalytic properties of ZnO nanoplatelets. Surf. Coat. Technol..

[31] Grüneberger F, Künniger T, Huch A, Zimmermann T, Arnold M (2015). Nanofibrillated cellulose in wood coatings: Dispersion and stabilization of ZnO as UV absorber. Prog. Org. Coat..

[32] Favarim HR, Leite LO (2018). Performance of ZnO nanoparticles for fire retardant and UV protection of pine wood. Bioresources.

[33] Ahn K, Zaccaron S, Zwirchmayr NS, Hettegger H, Hofinger A, Bacher M, Henniqes U, Hosoya T, Potthast A, Rosenau T (2019). Yellowing and brightness reversion of celluloses: CO or COOH, who is the culprit?. Cellulose.

[34] Michaelsen A, Pinzari F, Barbabietola N, Piñar G (2013). Monitoring the effects of different conservation treatments on paper-infecting fungi. Int. Biodeterior. Biodegrad..

[35] Shamsian A, Fata A, Mohajeri M, Ghazvini K (2006). Fungal contaminations in historical manuscripts at Astan Quds museum library, Mashhad, Iran. Int. J. Agric. Biol..

[36] Se J, He J (2021). Antimicrobial activity of Zinc oxide nano/microparticles and their combinations against pathogenic microorganisms for biomedical applications: From physicochemical characteristics to pharmacological aspects. Nanomaterials.

[37] Fu F, Li L, Liu L, Cai J, Zhang Y, Zhou J, Zhang L (2015). Construction of cellulose based ZnO nanocomposite films with antibacterial properties through one-step coagulation. ACS Appl. Mater. Interfaces.

